# Enhanced Thermo-optical Response by Means of Anapole Excitation

**DOI:** 10.1021/acs.jpclett.2c00870

**Published:** 2022-06-30

**Authors:** Javier González-Colsa, Juan D. Olarte-Plata, Fernando Bresme, Pablo Albella

**Affiliations:** † Group of Optics, Department of Applied Physics, University of Cantabria, 39005 Santander, Spain; ‡ Department of Chemistry, Molecular Sciences Research Hub, Imperial College London, London W12 0BZ, U.K.

## Abstract

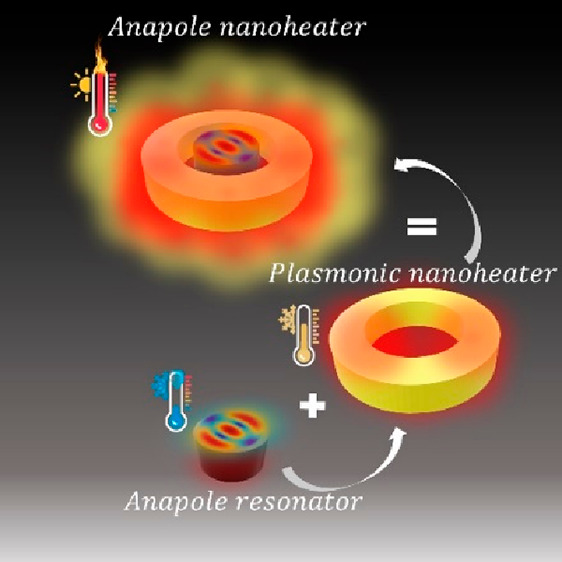

High refractive index (HRI) dielectric nanostructures offer a versatile platform to control the light–matter interaction at the nanoscale as they can easily support electric and magnetic modes with low losses. An additional property that makes them extraordinary is that they can support low radiative modes, so-called anapole modes. In this work, we propose a spectrally tunable anapole nanoheater based on the use of a dielectric anapole resonator. We show that a gold ring nanostructure, a priori nonresonant, can be turned into a resonant unit by just filling its hole with an HRI material supporting anapole modes, resulting in a more efficient nanoheater able to amplify the photothermal response of the bare nanoring. As proof of concept, we perform a detailed study of the thermoplasmonic response of a gold nanoring used as heating source and a silicon disk, designed to support anapole modes, located in its center acting as an anapolar resonator. Furthermore, we utilize the anapole excitation to easily shift the thermal response of these structures from the shortwave infrared range to the near-infrared range.

All-dielectric nanostructures based on high refractive index (HRI) materials offer the possibility to efficiently confine and manipulate light at the nanoscale.^1−3^ A wide variety of possible optical modes across the visible (VIS),^4^ near-infrared (NIR),^5^ and mid-infrared (MIR)^6^ ranges have been exploited in a wide variety of applications,^7^ such as electric and magnetic hot spot generation,^8^ strong metal/dielectric coupling,^9^ Purcell enhancement,^10^ highly directional scattering,^11^ or resonant absorption, among others. Conversely, plasmonic nanoparticles present high resistive losses enabling a rapid and remarkable temperature increment of their surroundings. This effect is enhanced at the plasmonic resonance, which is determined by the free-electron charge-density features (Frölich condition) and the particle shape and size. The sudden electron-density oscillations lead to an electric field enhancement that decays rapidly with the distance to the particle surface. These physical effects have been exploited in a wide range of applications, such as sensing,^12,13^ surface-enhanced Raman spectroscopy, surface-enhanced infrared absorption spectroscopy , or photoinduced heating,^14−19^ including photothermal therapies (PTTs).^20−22^ However, when the nanostructures become on the order of the excitation wavelength, the description of their electromagnetic response requires three multipolar series: the magnetic, electric, and toroidal.^23,24^ A toroidal dipole in combination with an electric one can produce a nonradiating charge current configuration known as a dynamic anapole.^25−29^ This state appears for a particular wavelength where the fields radiated by the toroidal and electric dipoles cancel each other via destructive interference. An ideal anapole excitation does not emit or absorb, and consequently, it cannot be detected in the far field. However, it results in a strong electric field enhancement. This enhancement has been used to develop and improve optical techniques such as Raman scattering,^30^ refractive index sensing,^31,32^ narrow band absorption,^33^ or even optothermal enhancement via lossy dielectrics.^34−36^ The study of anapole mode excitations has attracted interest recently, with current efforts directed to boost their efficiency beyond the near-field region. This enhanced efficiency can be achieved with single all-dielectric ring–disk structures. However, to the best of our knowledge, previous studies have disregarded the heating potential of metal–dielectric structures assisted by anapole modes, only focusing on the self-induced heating due to the low intrinsic absorption in HRI materials,^34^ that require excitation power densities that go beyond the conventional ones (up to 24 mW/μm^2^).

In this work, we propose an anapole nanoheater able to amplify the thermal response of a plasmonic nanoheater based on the use of a dielectric anapole resonator. This would allow the use of lower light intensities to achieve striking heating effects. To show this proof of concept, we consider a gold nanoring structure as heating source. This nanoparticle geometry is used since it provides enhanced heating.^20^ Then, we locate the HRI dielectric disk, designed to support anapole modes in its center. The thermal amplifying mechanism emerges from the excitation of the anapole mode that develops inside the nanoring (long lifetime mode^29^). The plasmonic ring surrounding the anapole resonator absorbs this local electric field, consequently suffering a strong amplification of its resistive losses and its conversion into heat. We also show that a plasmonic structure, a priori nonresonant, can be turned into an efficient resonant nanoheater by simply filling its hole with a HRI material that behaves as a dielectric anapole resonator. Therefore, high temperatures can be achieved in the NIR, a spectral region of particular interest in biomedical applications. We have used COMSOL Multiphysics to perform all the calculations in combination with Lumerical FDTD (finite difference time domain). The heat transport calculations were performed in two steps.^37,38^ The first consisted in solving the electromagnetic problem to obtain the volumetric distribution of the resistive losses; that is, we solved the Maxwell equations with the RF COMSOL suite. To do so, we illuminated the hybrid system with linearly polarized light, considering a free tetrahedral mesh with element sizes controlled by the excitation wavelength to guarantee a high element density and reliable curvatures when needed. On the other hand, to consider the heat dissipation, we used a heat flux node across the outer boundaries, considering a heat transfer coefficient, dependent on the geometry and the ambient conditions.

We have considered conduction as the main transfer mechanism as we are considering small structures in a stationary fluid. We also neglect the effect of the interfacial thermal conductance since in the steady state this parameter would increase the inner temperature without changing the external one; thus, similar fluid heating is expected. Also, the influence of the interfacial thermal conductance on the structure dynamics depends on the time pulse, which in our case is on the order of microseconds. Thus, a negligible effect of this parameter is expected.^39,40^ All the thermal properties involved in this study (density, specific heat, and thermal conductivity) were taken from the COMSOL Multiphysics material database.

Figure 1a depicts a schematic of the anapole excitation produced within a silicon disk of 340 nm radius when illuminated at the wavelength λ ≈ 1300 nm.^41^ The HRI disk scattering and absorption cross-sectional spectra are also shown in Figure 1a. Here, a spectral drop in the scattering cross section can be clearly seen for both materials together with null in the absorption cross section. It can be observed how the electric field lines follow the toroidal geometry shown in the upper inset. The electric field circulation produces the distinct anapole pattern (shown in the lower inset) that can be characterized by a sudden drop in the scattering spectra accompanied by a lack of absorption. These cross sections, together with the electric field spatial distribution (shown in the inset and Figure S1), evidence the excitation of a strong anapole mode.

**Figure 1:**
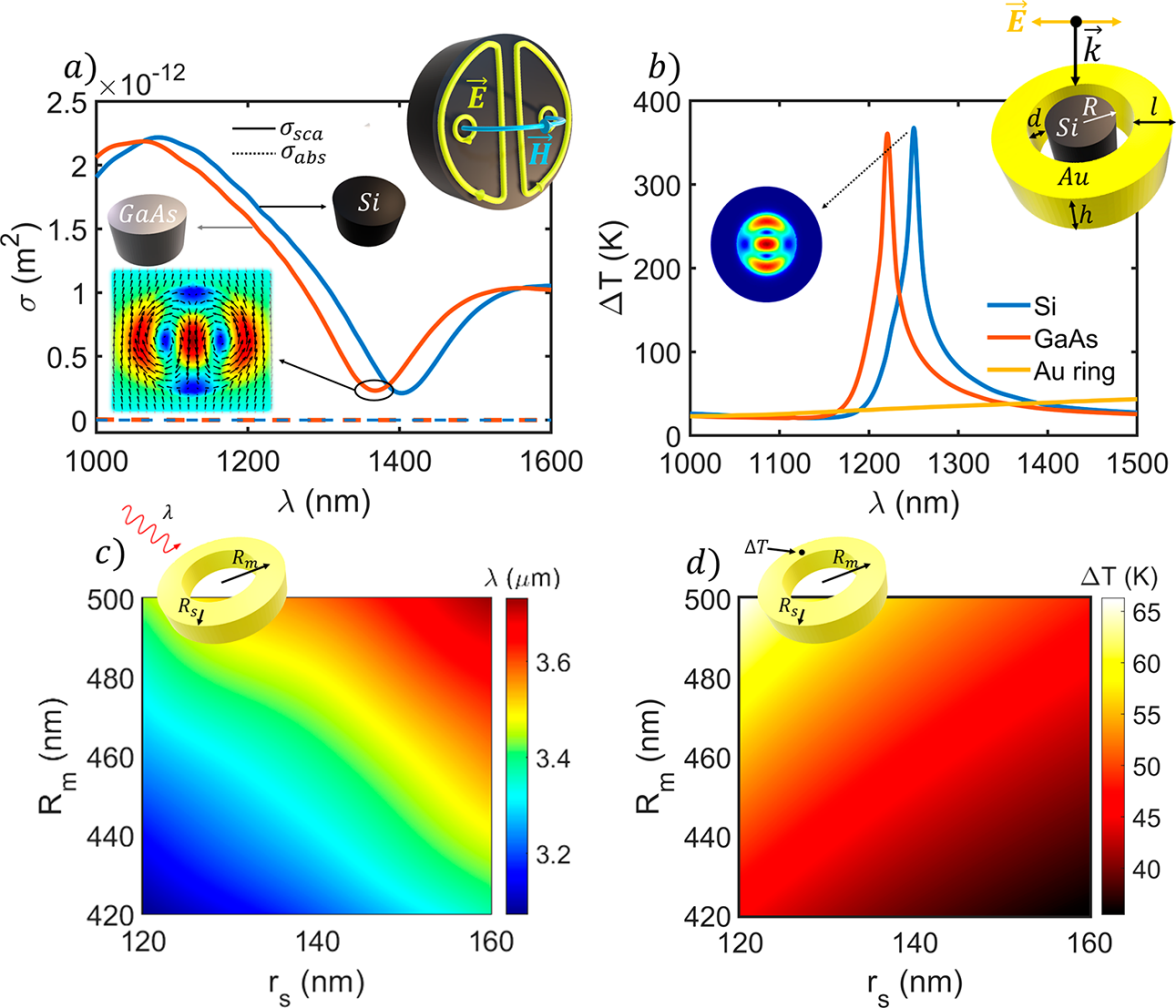
(a) Scattering (solid line) and absorption (dotted line) cross sections for silicon (blue) and gallium arsenide (orange) disks with *R* = 340 nm and *h* = 155 nm.^41^ Anapole mode electric field distribution (schematically represented in the inset). (b) Comparison of the spectral temperature increment distribution for the isolated gold ring (yellow) and the optimized anapole generators (outlined in the upper inset where *d* is the dielectric–metallic distance, *R* the disk radius, *l* the ring length, and *h* the height) defined by *d* = 0 nm, *R* = 340 nm, *l* = 290 nm, and *h* = 180 nm. The incident power density is fixed at 0.1 mW/μm^2^. (c and d) Color maps of (c) the thermoplasmonic spectral resonance and (d) the maximum temperature increase at that wavelength for a set of gold nanoring designs defined by a main radius (*R*_*m*_) from 420 to 500 nm and a secondary radius (*r*_*s*_) from 120 to 160 nm.

Furthermore, their cross sections also reveal that the anapole mode spectrally differs for Si and GaAs disks, with the response in the case of silicon red-shifted with respect to that of gallium arsenide. This is a consequence of their slightly different optical constants. Besides that, materials such as Si or GaAs have a very low imaginary part of the refractive index in the NIR and MIR translating into a negligible absorption. The optical response of the system is determined by the optical properties (dielectric function) and shape of the structure. In the case of an anapole (dipolar + toroidal modes), it is the dipolar one that has the main contribution to its spectral width (see ref (42)). Figure 1b illustrates the analyzed hybrid nanostructure built by a metallic nanoring (made of gold) and the HRI disks shown in Figure 1a (two different materials are explored, Si and GaAs). The dimensions of the hybrid disk/ring structure described before are not arbitrary but those that provide maximum absorption and, consequently, maximum heat. These dimensions, ring width (*l*), height (*h*), and metal–dielectric distance (*d*), are the result of a study on the influence that the ring size parameters have on the electromagnetic system response (see Figures S2–S6 of the Supporting Information for more details). The results show that the ring parameters mainly influence the ability of the nanomaterial to reach high temperatures. The optimal heating response is reached for full contact between both materials, *d* = 0 nm, since the resistive loss is enhanced as gold approaches the HRI resonator (see Figures S2 and S3). Although fabrication of these kinds of systems may be challenging, complex structures can be made by high resolution techniques such as e-beam lithography,^43^ sequential lithographic deposition/etchings for each material,^44^ and critical energy electron beam lithography.^45^ Once the ring parameters had been analyzed, the influence of *h* on the thermoplasmonic response of the hybrid system was investigated. It is found that the highest temperature increase is obtained for *h* = 180 nm (see an extended discussion in Figure S5).

In Figure 1b the thermal spectral response of the optimal hybrid system is also compared with an isolated ring showing that the ring thermal response is more than 10 times lower than the hybrid one. This can be explained by the anapole excitation which boosts the ring electromagnetic response acting as the electromagnetic resonator. This enhanced electric field induces a more intense Joule effect leading to an increment of the resistive losses. In particular, the maximum temperature increment is found for the excitation wavelengths λ ≈ 1250 nm and λ ≈ 1213 nm, for the Si and GaAs disks, respectively. The input power density is 0.1 mW/μm^2^. The low absorption in the disk resonator is an important characteristic, since highly absorptive materials reduce the anapole efficiency (the electric energy is consumed by the HRI material) affecting the resistive losses in the gold ring. This translates in a weaker heating (the temporal evolution of the electromagnetic resonance is shown in Movies S1, S2, and S3).

A detailed analysis of the thermoplasmonic response of metallic rings with similar sizes to the ones of our design was performed to fairly compare them. For this purpose, three parameters should be considered: the resonance spectral region, the maximum temperature increase, and the heated volume. The results are shown in Figure 1c and d (see Figures S7–S9 to visualize a more extended analysis). In this figure, two color maps corresponding to the spectral response of a single ring and the maximum temperature increment obtained at those wavelengths are depicted. As can be seen, when increasing the main and secondary radii, the ring resonance shifts to longer wavelengths, making impossible its direct comparison with the hybrid one at the same spectral regions. Once the dependence of the nanomaterial geometry on heating has been analyzed, we focus on the spectral tunability of the structure. Thus, two of the most relevant parameters are investigated: disk radius and structure height.

As shown in Figure 2a and b, variations of the disk radius and structure height give rise to remarkable changes in the thermal spectrum (see the analogue analysis for the GaAs disk in Figure S10). The anapole mode is red-shifted for larger radii and heights, as expected. This supports the notion that a hybrid disk/ring resonator can be easily tuned within the metal–dielectric, where a ring-shaped gold nanostructure of the same size is unable to resonate.^46,47^ An interesting aspect shown in Figure 2a and b is the presence of two different resonances. After a detailed thermoplasmonic analysis of the system (see Figure S11 for more information), we attribute this splitting to the presence of two different anapolar modes. The stronger heating mode corresponds to the aforementioned anapole excitation which is attributed to an electric anapole. However, based on the electric and magnetic near-field distributions shown in Figure S11, the lower heating peak seems to correspond to a magnetic anapole.^48,49^ Notice that the double spectral resonances overlap as radius approaches 340 nm and height approaches 180 nm, giving the optimum configuration in terms of the maximum temperature increase.

**Figure 2:**
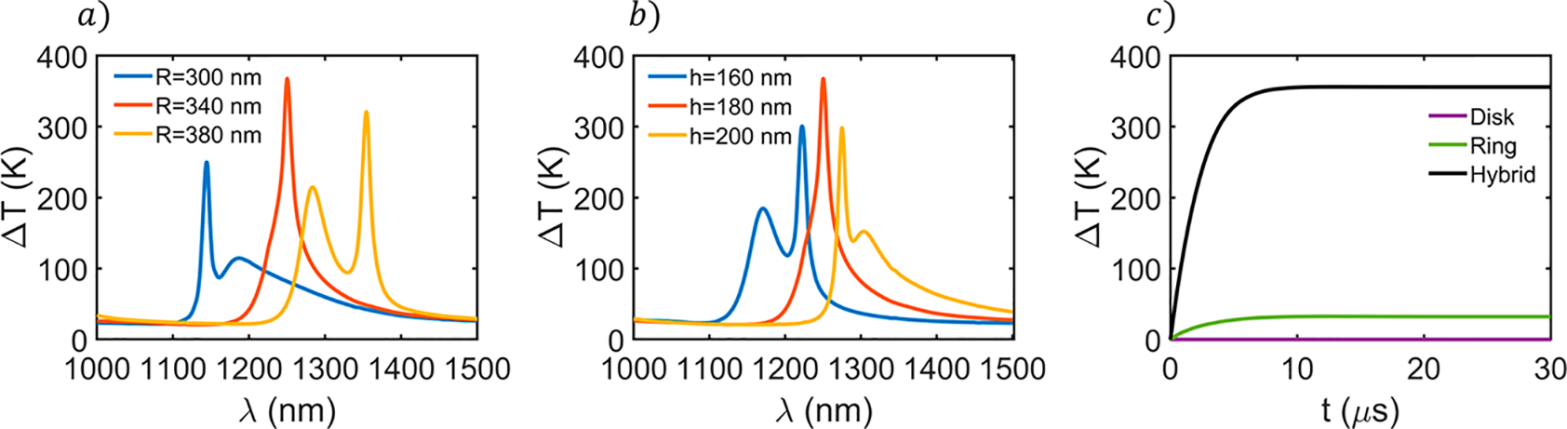
(a and b) Spectral thermal response of the optimal hybrid ring/disk resonator calculated for different (a) silicon disk radii and (b) structure heights. The ring length and metal–dielectric distance are fixed to *l* = 290 nm and *d* = 0 nm, respectively. (c) Transient thermal state for the isolated silicon disk (purple), ring (green), and hybrid structure (black) when illuminated at λ ≈ 1250 nm.

The transient thermal state is calculated for the isolated ring and disk compared with the hybrid unit cell to get the heating time scale of the systems. Thus, as can be seen in Figure 2c), the temperature increase is negligible in the case of the isolated silicon disk. This can be attributed to the lack of absorption that silicon shows within the NIR. Despite the fact that the ring-shaped and hybrid structures present remarkable differences in temperature, it can be seen that they reach the stationary state for similar periods of around ∼5 μs. This means that the system would have a threshold excitation pulse time in terms of heating. For example, the structure would see a nanosecond laser as a continuous wave laser since the heating process is too slow compared with the laser repetition rate. Conversely, if we think about a microsecond laser, probably the system will suffer an oscillatory thermal response, which may affect the thermal efficacy for a specific application. We have presented above a comprehensive study of the hybrid disk–ring performance as a heating unit. We now explore its behavior when a substrate is present. To do so, we consider the configuration defined by *R* = 340 nm, *l* = 290 nm, *d* = 0 nm, and *h* = 180 nm. Commonly used substrates with contrasting thermal properties have been considered to demonstrate the huge impact that the thermal conductivity has on the photothermal capabilities of the proposed structure. We have selected alumina, silica, and polydimethylsiloxane (PDMS) as examples of high, middle, and low thermal conductivities with 24 W/mK, 1.3 W/mK, and 0.15 W/mK, respectively.

In Figure 3a, the scheme of the hybrid structure on a substrate of thermal conductivity *k* can be seen. We consider a power density of 0.1 mW/μm^2^ and linearly polarized light to excite the hybrid structure at normal incidence. In Figure 3b, the spectral thermal responses of the three substrates are shown. All curves show the spectral resonances at similar wavelengths as those of the considered materials having similar optical properties (the influence of the substrate refractive index on the architecture cross sections is shown in Figure S12). However, they present radically opposed thermal responses as a consequence of their thermal conductivity contrast. A well-defined trend can be extracted from the maximum temperature increment.

**Figure 3:**
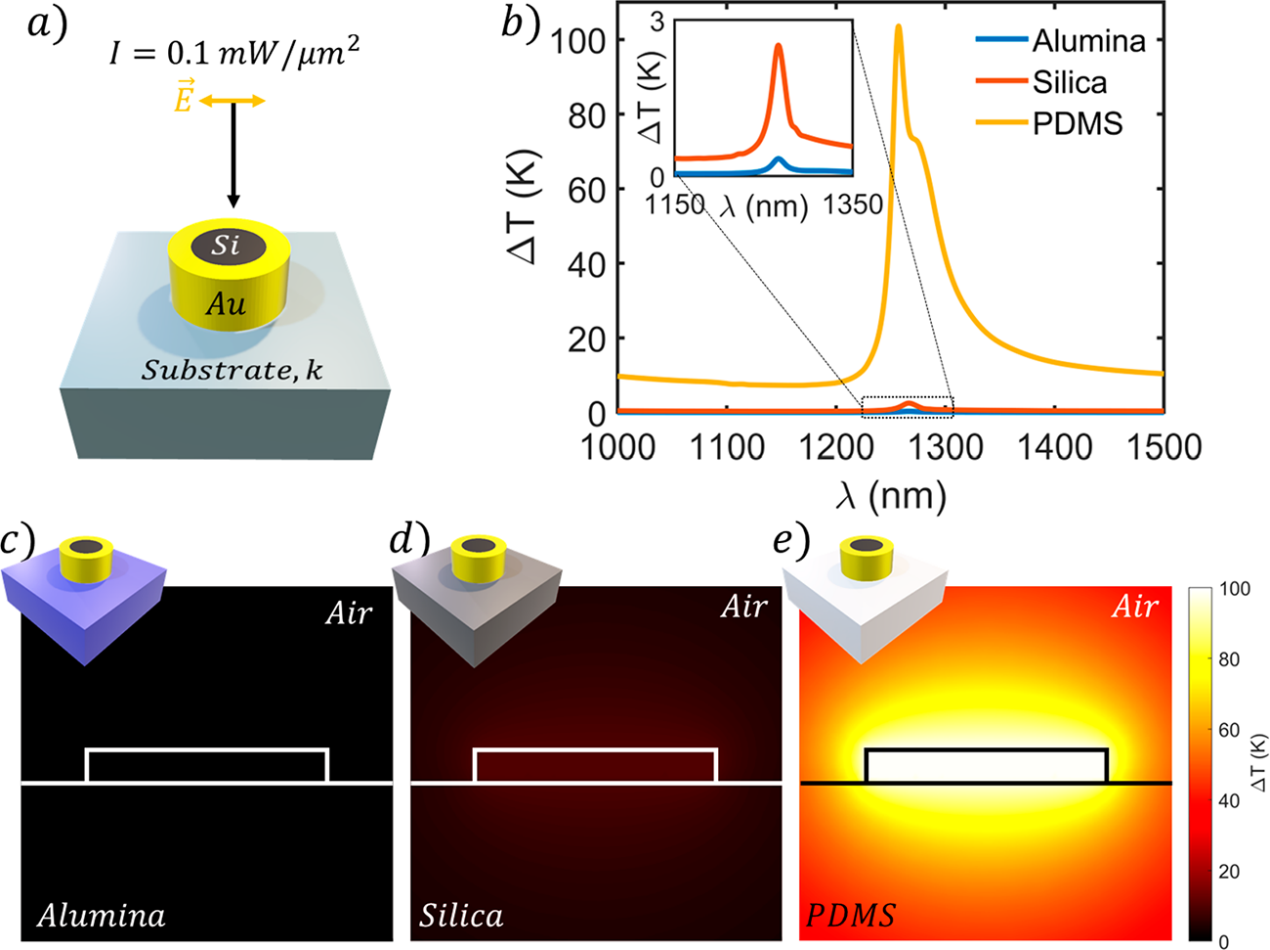
Thermal behavior of the proposed structure on different conductive substrates. (a) Scheme of the hybrid structure on a substrate of thermal conductivity *k* illuminated at normal incidence with a power density of 0.1 mW/μm^2^. (b) Temperature increment spectra for the three considered materials, i.e., alumina (blue), silica (red), and PDMS (yellow). (d–f) Temperature increment spatial distribution for a substrate of (d) alumina, (e) silica, or (f) PDMS.

As the substrate thermal conductivity grows, the temperature decreases. The temperature is also smaller with respect to the isolated hybrid structure, as air has a much lower thermal conductivity. Thus, the PDMS substrate shows the best performance, allowing for temperature increments of about 100 K. Following Figure 3c–e, the alumina substrate features a negligible heating effect and the silica substrate is more than 90% less effective than the PDMS one. This result can be understood by considering the high conductivity of the substrates and how the heat flows preferentially through the higher thermal conductivity materials, so that the stationary temperature increment is reduced significantly. Conversely, low thermal conductivity materials inhibit heat flow, leading to a higher increase of the nanomaterial temperature in the stationary state (see Figure S13 to visualize the analogue analysis for the GaAs disk).

In summary, we have demonstrated that anapole excitation can serve as an easy mechanism to boost the heating effect in ring-shaped gold structures. Additionally, their thermal response can be tuned to the NIR, where they are not able to resonate a priori. Their thermal performance was analyzed by placing them on different thermal conductivity substrates (alumina, silica, and PDMS), obtaining temperature increments 10-fold that of single gold rings. We believe that the reliable implementation of our proof of concept can motive the development of novel strategies to reach efficient nanoheating structures and temperature-controlled platforms that will be of general interest to the thermoplasmonic community.

## Supporting Information Available

Additional computational details, materials, and descriptions, including the optimization of the proposed anopole nanoheater (PDF)

## Notes

Movies of the temporal evolution of the electromagnetic resonance are available in the online version of the paper.
