# Supplementary material for: Enhanced Thermo-optical Response by Means of Anapole Excitation
Source: J Phys Chem Lett. 2022 Jun 30;13(26):6230–5. doi: 10.1021/acs.jpclett.2c00870 (PMC9272441; doi:10.1021/acs.jpclett.2c00870)
Supplement: Supplementary file 1 — jz2c00870_si_001.pdf [file jz2c00870_si_001.pdf]

Enhanced Thermo-Optical Response by Means of Anapole Excitation

Javier González-Colsa[1], Juan D. Olarte-Plata[2], Fernando Bresme[2] and Pablo Albella[1,]*

[1]*Group of optics, Department of Applied Physics, University of Cantabria, 39005, Santander, Spain.*
[2]*Department of Chemistry, Molecular Sciences Research Hub, Imperial College London, W12 0BZ UK.*

*pablo.albella@unican.es

Supplementary document

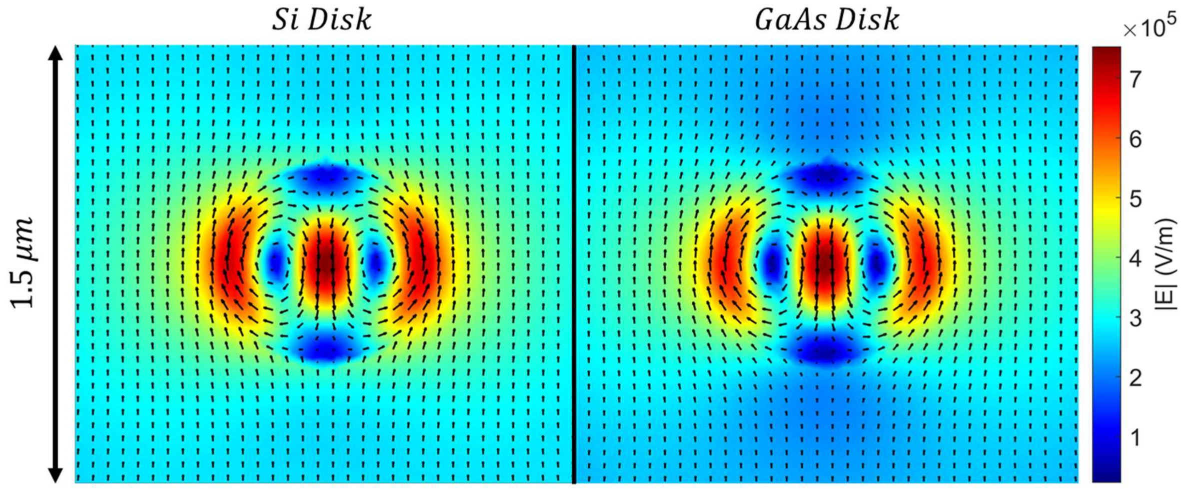

Figure S1. Electric field distributions of the resultant anapole mode excitation in silicon and gallium arsenide disks at λ =1400 nm and λ =1366 nm. The disk is immersed in air and has a height h = 155 nm and a radius R = 340 nm.

Figure S1 shows the electric field distribution of the anapole modes induced within a silicon and gallium arsenide disks excited at a wavelength of 1400 nm and 1366 nm respectively. The resonance wavelength is slightly different due to the refractive index contrast between both materials. Thus, both systems show similar electric field pattern and lines that match with the expected ones for an anapole mode excitation.

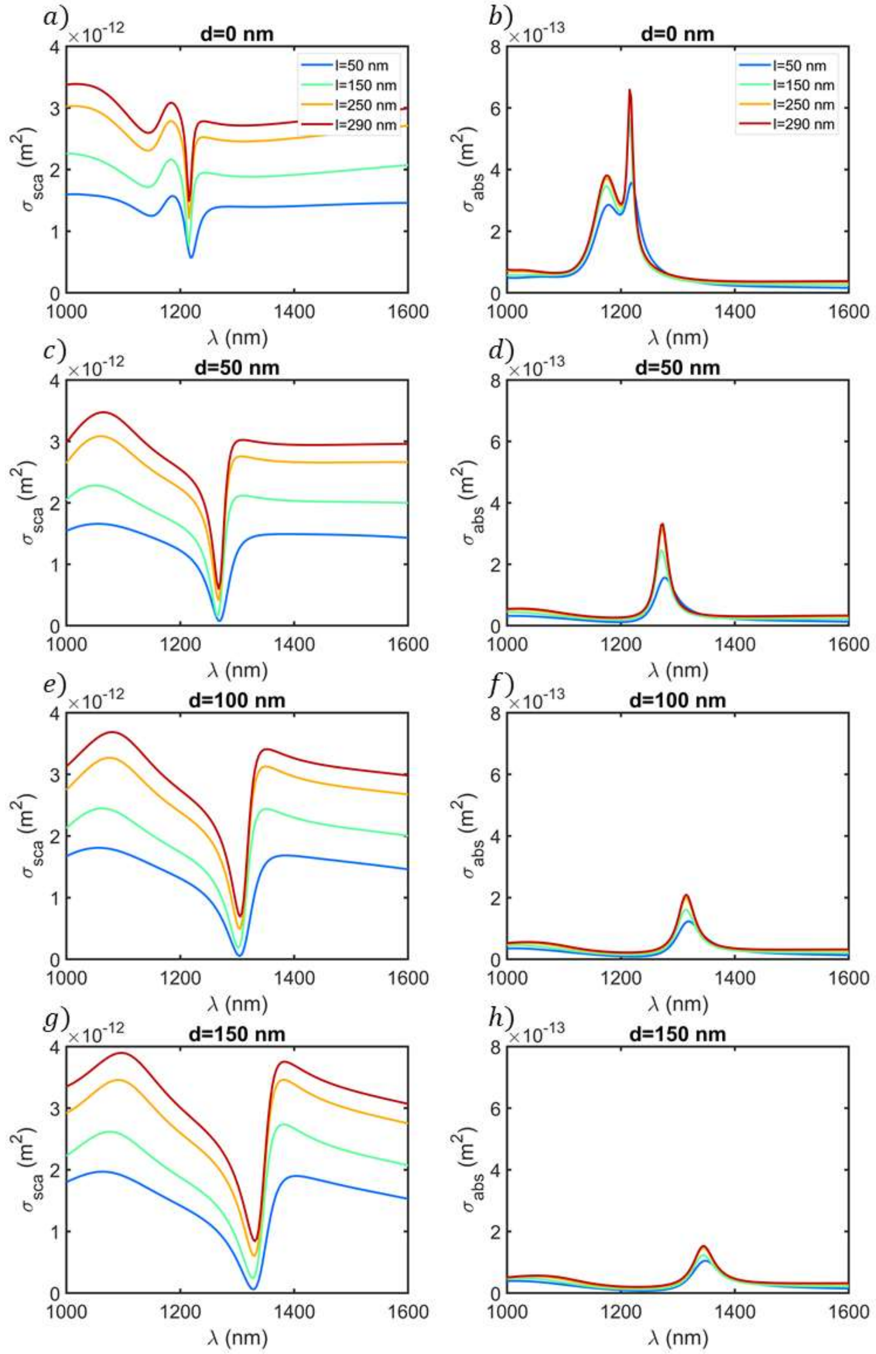

Figure S2. Scattering and absorption cross section of a hybrid silicon-gold disk/ring structure for a set of ring lengths (l) from 50 to 290 nm for $d = 0\ nm$ (a, b), $d = 50\ nm$ (c, d), $d = 100\ nm$ (e, f) and $d = 150\ nm$ (g, h), respectively.

In Figure S2 the behavior of the hybrid silicon/gold structure absorption and scattering cross sections can be seen. In general, the scattering (Figure a, c, e, g) increases for thicker gold rings (increasing l) as expected, however, the spectral resonance remains almost constant. The l parameter also boosts the absorption due to the gold volume increment. Focusing on the absorption (Figure b, d, f, h), the most influential geometrical parameter is the metal-dielectric distance. As d goes to zero the absorption increases which is translated in a thermal performance improvement. This can be explained in terms of the anapole resonance. When the electric anapole is generated inside the disk, an evanescent field is created in the near field so that, the nearer the gold is to the dielectric interface, the stronger the evanescent field influence becomes. This leads to a resistive loss enhancement in the metal. It is also noticed a moderate blueshift because of the ring main radius reduction. Thus, in sight of Figure S2, the optimal disk-ring configuration in terms of light to heat conversion is $d = 0\ nm$ and $l = 290\ nm$.

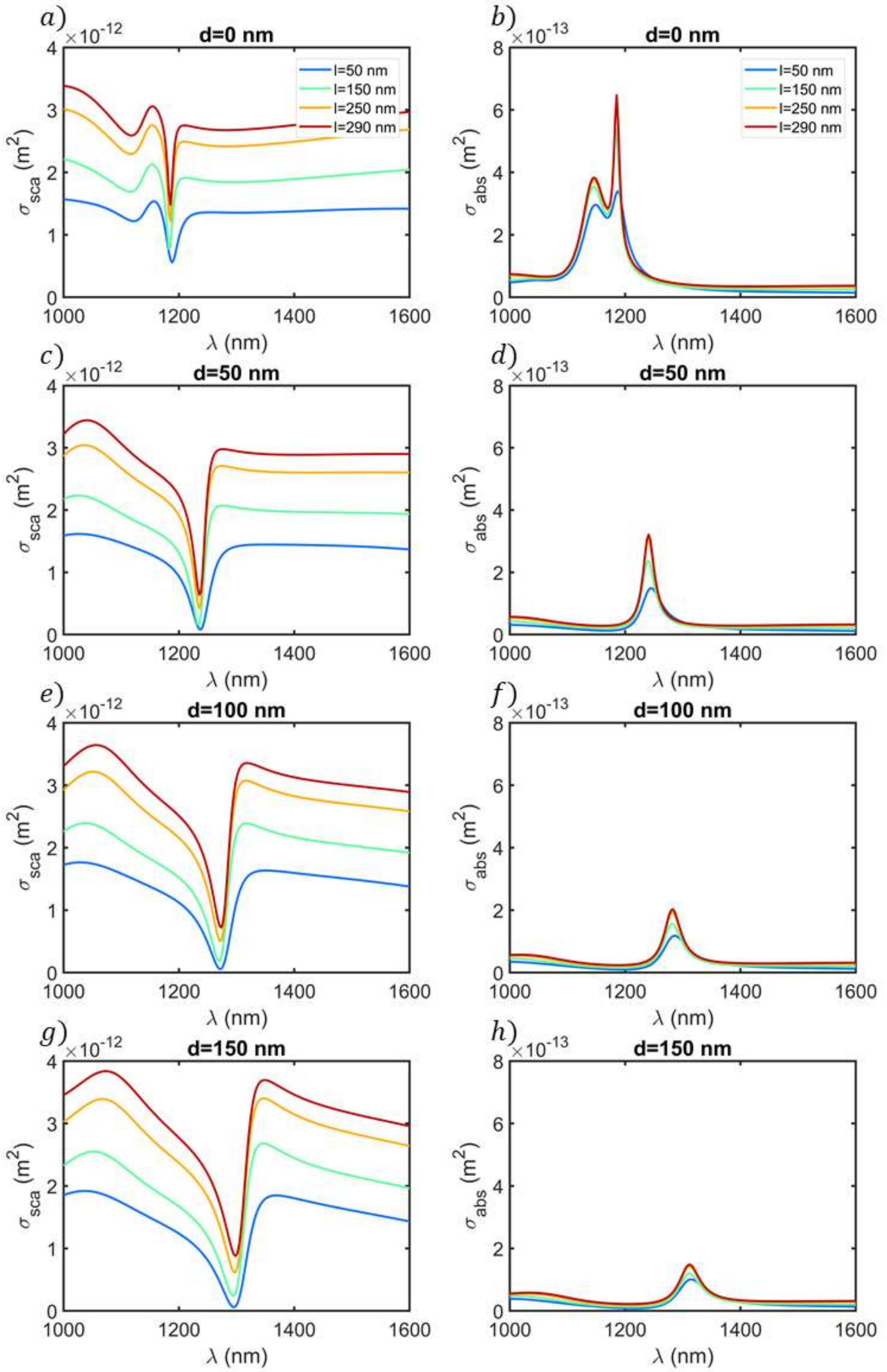

Figure S3. Scattering and absorption cross section of a hybrid gallium arsenide-gold disk/ring structure for a set of ring lengths (l) from 50 to 290 nm for $d = 0\ nm$ (a, b), $d = 50\ nm$ (c, d), $d = 100\ nm$ (e, f) and $d = 150\ nm$ (g, h) respectively.

In Figure S3 the behavior of the hybrid gallium arsenide/gold structure absorption and scattering cross section can be seen. It shows a similar behavior to the Figure S2 so that the same conclusions are extracted. Thus, the optimal disk-ring configuration in terms of light to heat conversion is also $d = 0\ nm$ and $l = 290\ nm$.

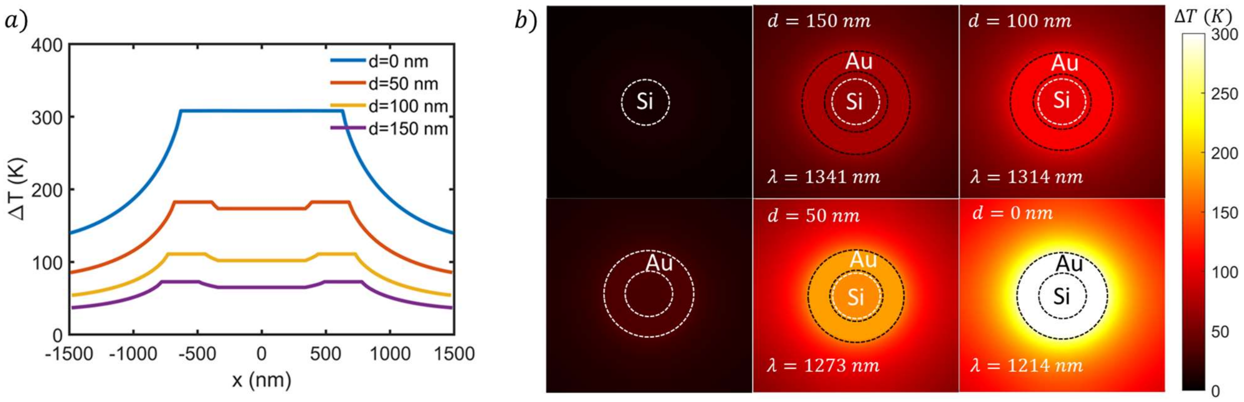

Figure S4. a) Temperature profile of the studied hybrid structure for $l = 290\ nm$, $h = 155\ nm$, R=340 nm and different d values from 0 to 150 nm. b) Temperature maps for the same conditions in a). The map size is 3 μm.

In Figure S4a the temperature profiles for the hybrid gold/silicon structure are shown. They have been taken on the x-axis, i.e., a line passing through the center of the structure. The system parameters have been fixed to l=290 nm, h=155 nm and R=340 nm. It can be seen that the maximum temperature decreases when the gold/dielectric distance increases. This can be explained in terms of the anapole influence on the gold ring. As gold approaches the dielectric surface, the interaction of the anapole electric field and the gold free electrons gets stronger, leading to a resistive loss intensification. Thus, a temperature enhancement is produced. In Figure S4b the temperature maps corresponding to those metal-dielectric distances can be visualized. Notice that the temperature increase of the isolated dielectric disk is zero as the imaginary part of the silicon refractive index is negligible. Therefore, the resistive losses are produced in the metallic material (gold in this case). Then, in the hybrid configuration both temperatures, the dielectric and

gold ones are equalized for the full contact configuration so that the heat transfer by conduction is intensified.

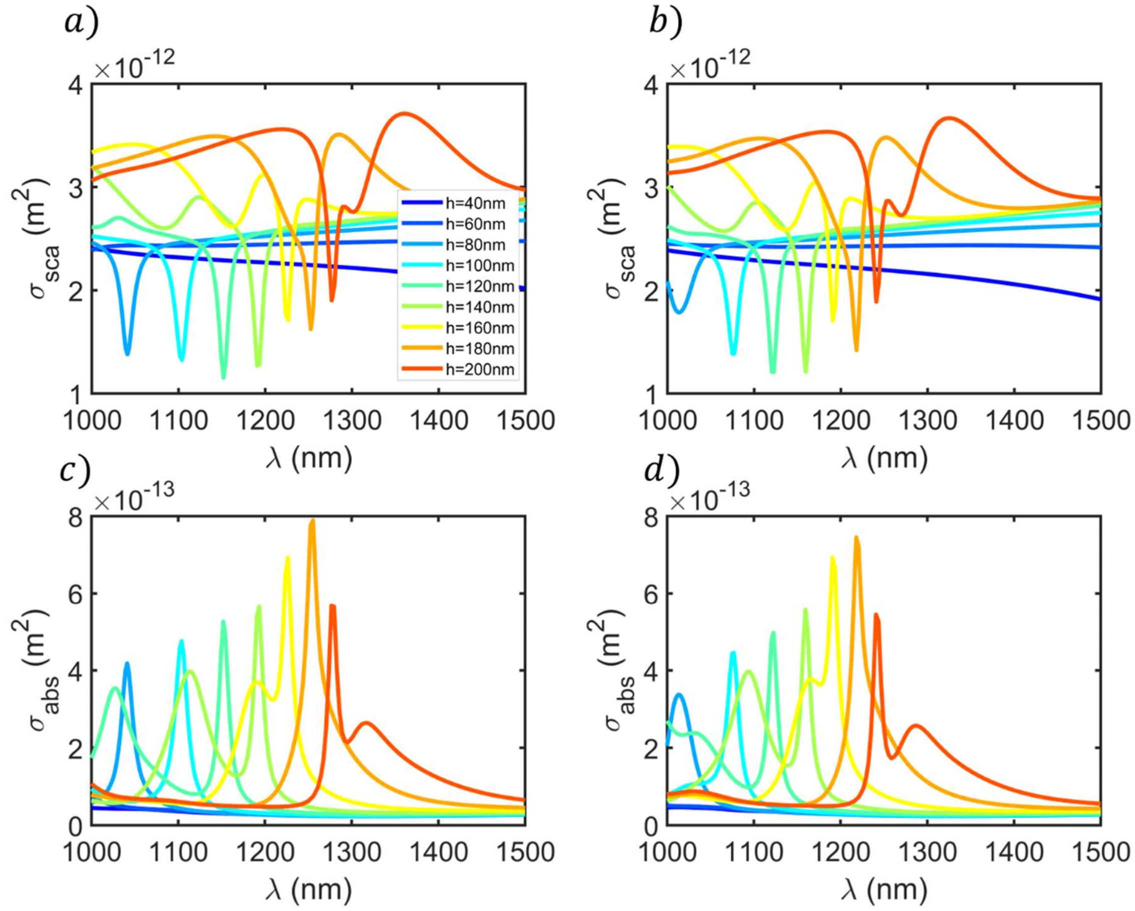

Figure S5. Scattering and absorption cross section of a)-c) a silicon and b)-d) gallium arsenide -gold disk/ring structure for a set of ring heights (h) from 40 to 200 nm for $d = 0\ nm$ and $l = 290\ nm$.

Figure S5 shows the scattering and absorption cross sections for a set of hybrid HRI Dielectric Disk/ring nanoheaters, a)-c) silicon and b)-d) gallium arsenide. As can be seen, two different resonances can be generated, the most intense corresponds to the electric anapole and the other seems to correspond to a magnetic one (see Figure S12). The most interesting feature in Figure S4 is the spectral shift suffered by both resonances for h variations. This could be explained in terms of the displacement currents produced inside the HRI disk. To have an anapole, there must be a

toroidal mode that comes out from the circulating displacement currents along the height of the disk. Larger disk heights require longer wavelengths as to form this circulating current.

In the case of the electric anapole, these currents are mostly contained in the plane of the disk. This means that as the height increases, the volume increases, thus increasing the excitation wavelength. However, in the case of the magnetic anapole, the displacement currents are mostly contained in the plane orthogonal to the plane of the disk, making it more sensitive to variations in height. This can explain the fact that the magnetic anapole is more sensitive to height variations than the electric one.

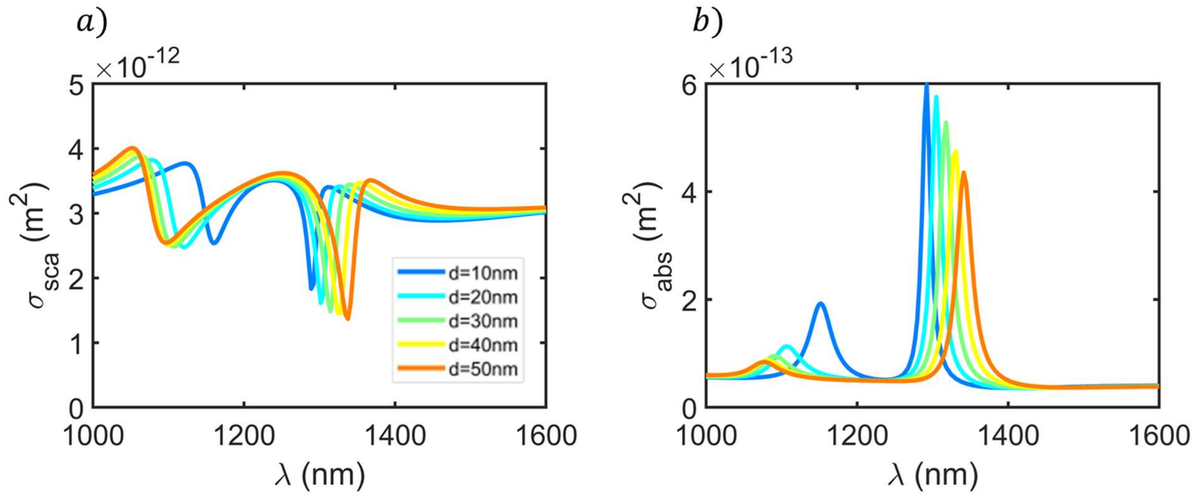

Figure S6. Scattering (a) and absorption (b) cross-sections of a hybrid disk-ring structure defined by $R = 340\ nm$, $l = 290\ nm$,$h = 180\ nm$ as a function of the metal-dielectric distance d.

Figure S6 shows the cross sections of the optical hybrid structure taken from the height optimization in Figure S5. The structure is characterized by $R = 340\ nm$, $l = 290\ nm$, $h = 180\ nm$ and its response to metal-dielectric distance is shown. Both scattering and absorption show the two anapolar modes. The most remarkable aspect to consider in our thermal analysis is that the absorption of the system increases as d reduces. This, again, can be understood in terms

of the electromagnetic interaction between the electric field generated in the disk with the gold ring free electrons, leading to an enhancement of the ring resistive losses. If we look at the electric anapolar mode we can see that it suffers a blue-shift in wavelength as we decrease *d*. This could be explained from the way we vary *d*. To decrease *d*, we keep the nanoresonator geometry (disk) fixed and reduce the main radius of the ring. Therefore, the electromagnetic response of the ring at that spectral region will correspond to a shorter wavelength each time. On the other hand, the magnetic anapole undergoes a redshift that also appears to be nonlinear when decreasing *d*. This could be understood in terms of the metal-dielectric interaction, which increases as the walls of the metallic ring approach the disk.

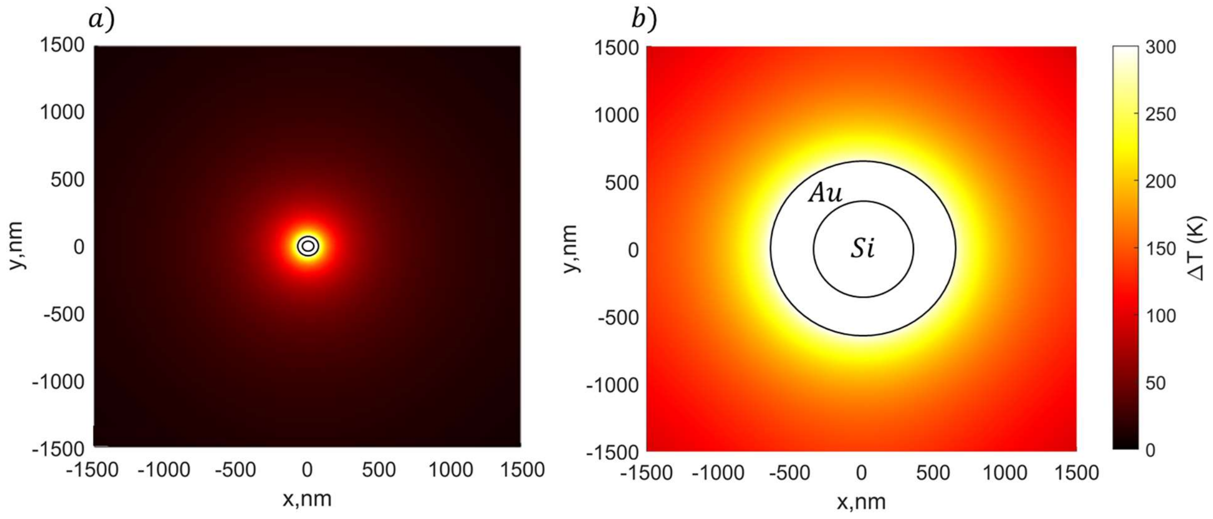

Figure S7. Temperature maps of the toroid (left) and hybrid anapolar nanoheater (right) excited at the resonance wavelengths: $\lambda = 900$ nm and $\lambda = 1250$ nm, respectively.

Figure S7 shows a comparison of the spatial temperature distributions of a metallic toroid (defined by the main and the secondary radius with values of R=50 nm and r=10 nm, respectively) and our anapolar hybrid resonator. Here, it can be seen how the hybrid structure heats up a significantly

larger volume. This also supports the idea that it is necessary to consider structures not only geometrically equivalent, but also comparable in size to allow for a fair comparison. To get an idea, we show that the toroid immersed in air can increase the temperature up to 400K, considering that there are no phase changes. The anapolar hybrid structure shows a temperature increase of 370K. Therefore, we can see that both temperatures are of the same order of magnitude. However, we must remember that the maximum temperature is that reached at the surface of the metal, so this data by itself does not incorporate the information on how this structure heats the surrounding medium.

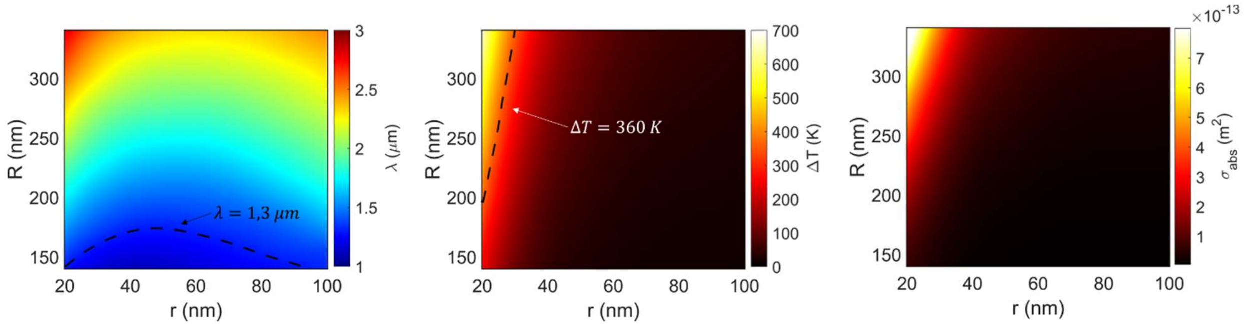

Figure S8. Thermoplasmonic optimization of a gold ring illuminated with an optical power density of 0.1 mW/μm^2. Color maps of a) the resonance wavelength, b) temperature increment at the resonance and c) absorption cross section. Dotted lines represent the spectral and thermal regions containing the thermoplasmonic state of the optimum anapole resonator: $R = 340\,nm$, $l = 290\,nm$, $d = 0\,nm$ and $h = 180\,nm$.

Figure S8 shows the optimum thermoplasmonic response of a set of gold nanorings defined by their main (R_m) and secondary (R_s) radii. These radii have been taken from 140 to 340 nm and from 20 to 100 nm, respectively. As shown in Figure S8a, the gold ring resonance is redshifted as the main radius increases. This can be explained in terms of a strong dipolar excitation involving the entire ring perimeter. The equal sign charges at opposite sides of the ring wall interact with a

strength determined by the ring thickness l. Thus, the distance between opposite sides increases with main radius making larger the dipole distance and leading to the mentioned spectral redshift.

On the other hand, the behavior of the spectral resonance shows a similar trend with respect to secondary radius variations up to a certain aspect ratio l/R (limited by about 50 nm secondary radius in our structure). In that case, the reduction of the secondary radius translates in an increased distance between the opposite ring sides, promoting the observed redshift. However, for larger aspect ratios, the ring hole is continuously decreased so that the geometry tends to that of a disk. Furthermore, this implies that both ring sides are closer, thus promoting their interaction and leading to the observed blueshift. In Figure S8b, the surface temperature increment of the ring is shown as a function of the geometrical parameters. It shows a clear trend: the temperature increment reduces for bigger secondary radii and increases with the main radius. This trend can also be observed in the absorption cross section (Figure S8c) which explains the ring temperature increment and demonstrates that both magnitudes are almost proportional. Therefore, from Figure S8, it is suggested that the temperature increment of the ring-shaped structures considered in our investigation can be qualitatively estimated from their absorption. Thus, absorption can be taken as a merit function to optimize the temperature increment of this structure. Besides, regarding the spectral location of the anapole nanoheater presented in this work ($\lambda \approx 1250\, nm$), it can be seen that there is not a ring-shaped structure able to reach temperature increments of about $\Delta T \approx 360$ K for comparable rings sizes, i.e., parameters close to $R = 340\, nm$, $l = 290\, nm$ and $h = 180\, nm$.

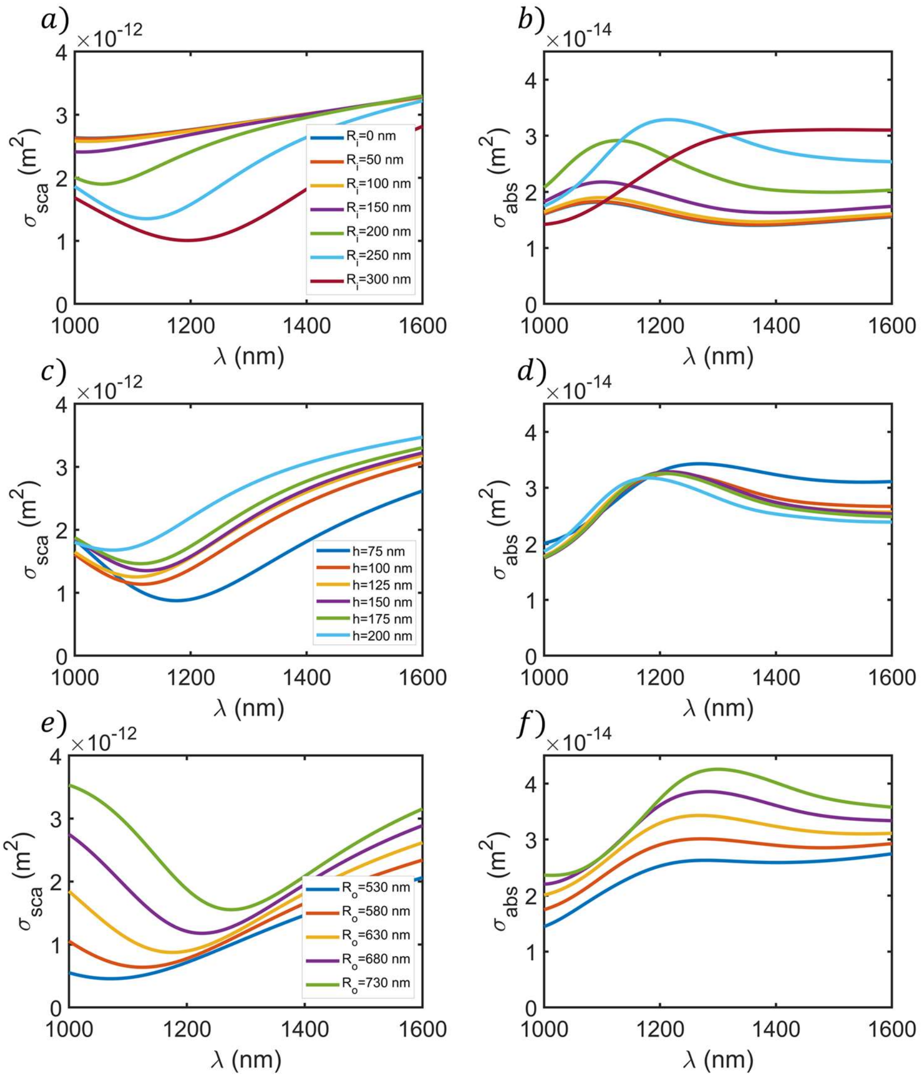

Figure S9. Scattering and absorption cross section of a gold nanoring structure for a set of ring inner radii (R_i) from 50 to 300 nm (a, b) for heights d from 75 to 200 nm (c, d), and outer radii R_o from 530 to 730 nm (e, f).

Figure S9 shows the scattering and absorption cross sections for a set of different isolated gold nanorings. In Figure S9a-b the ring height and outer radius were fixed to $h = 155\ nm$ and $R_o = 630\ nm$. It can be noticed that the absorption grows for increasing inner radius while being slightly redshifted and so does the scattering cross section. In Figure S9c-d, the starting point was the most absorptive configuration, that corresponds to an inner radius of $R_i = 250\ nm$ and outer radius of

$R_o = 630\ nm$. The variations in height reveal a different behavior. The spectra are blueshifted for growing heights while keeping the maximum absorption almost invariant. The spectral shift can be understood in terms of the geometry which is getting closer to a cylinder. Finally, Figure S9e-f show a similar trend than in the case of inner radius variations, once fixed the most beneficial structure in terms of absorption. Thus, the partially optimized gold ring is defined by $R_i = 250\ nm$, $R_o = 730\ nm$, and $h = 75\ nm$.

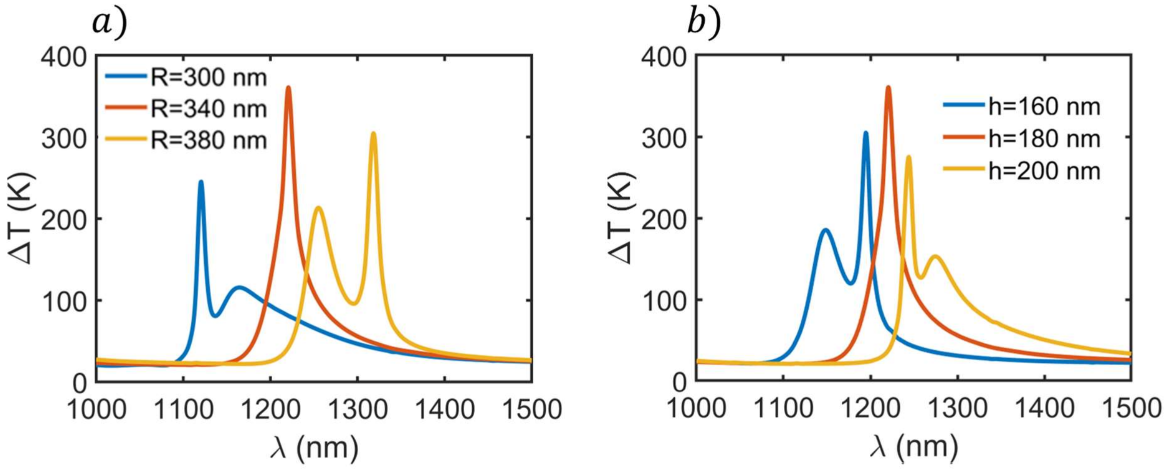

Figure S10. Temperature increase of the optimal gold/GaAs hybrid nanoheater for disk radius (a) and height (b) variations.

Figure S10a-b shows as an example, the thermal spectra of another high refractive index dielectric anapolar resonator (GaAs) that can alternatively be used in the hybrid structure, instead of the one in the main text (Si), under disk radius and structure height variations. This gives rise to remarkable changes in the thermal spectrum similar to those exposed in the main text. The anapole mode is also redshifted for larger radii and heights, as expected.

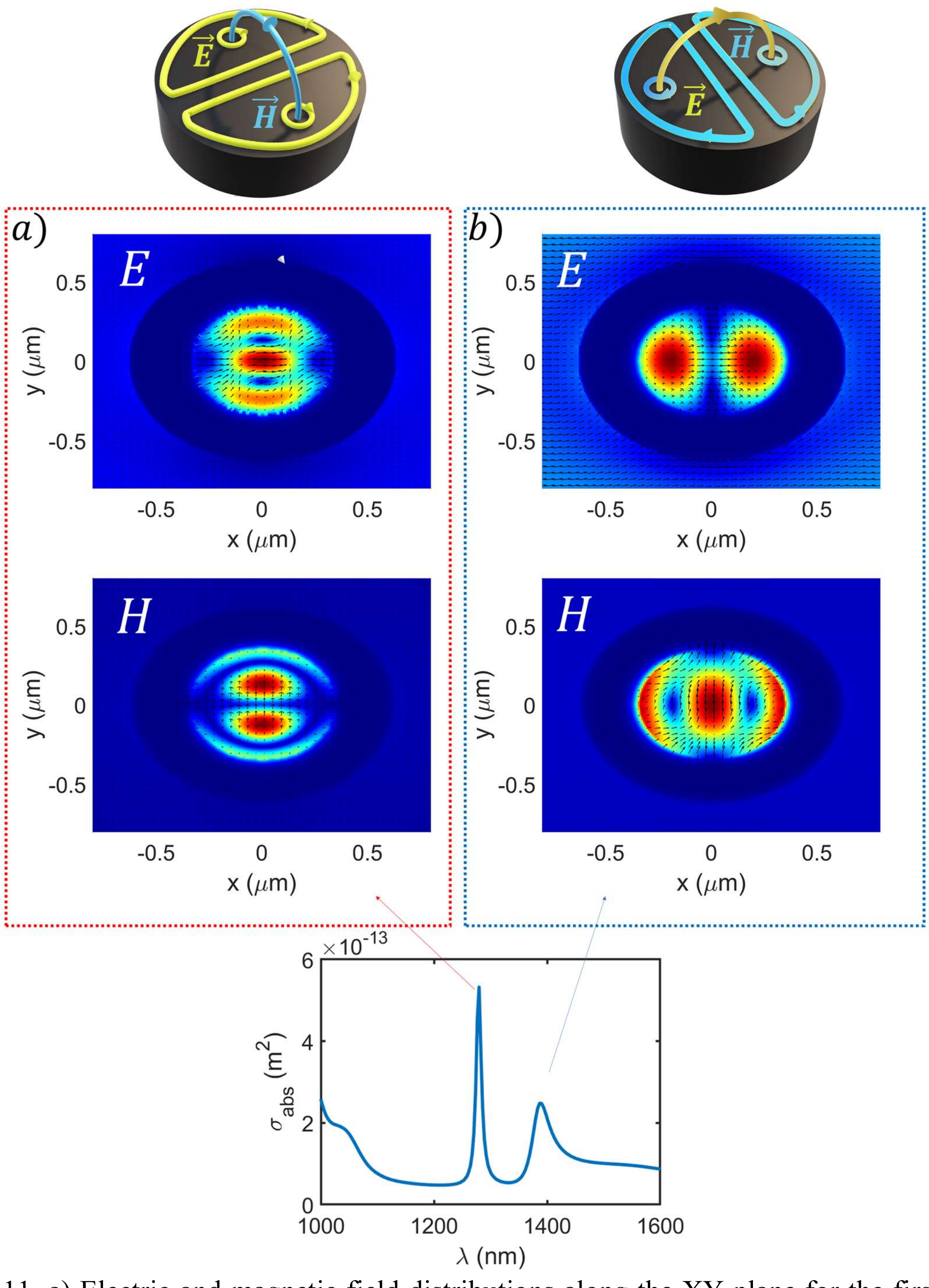

Figure S11. a) Electric and magnetic field distributions along the XY plane for the first (a) and second (b) modes. Lower inset: absorption cross section of a hybrid disk/ring nanoheater defined by R=340 nm, l=290 nm, d=0 nm and h=240 nm.

Figure S11a and S11b show the electric and magnetic field XY maps for each resonance. In the main text we just focused on the main resonance which corresponded to an electric anapolar mode (confirmed by the well-known characteristic shape of the E and H fields and reported in a big set

of references cited in the main text). However, in view of the spatial distribution of the electric and magnetic fields of the second, it seems to correspond to an anapolar resonance, but this time magnetic.

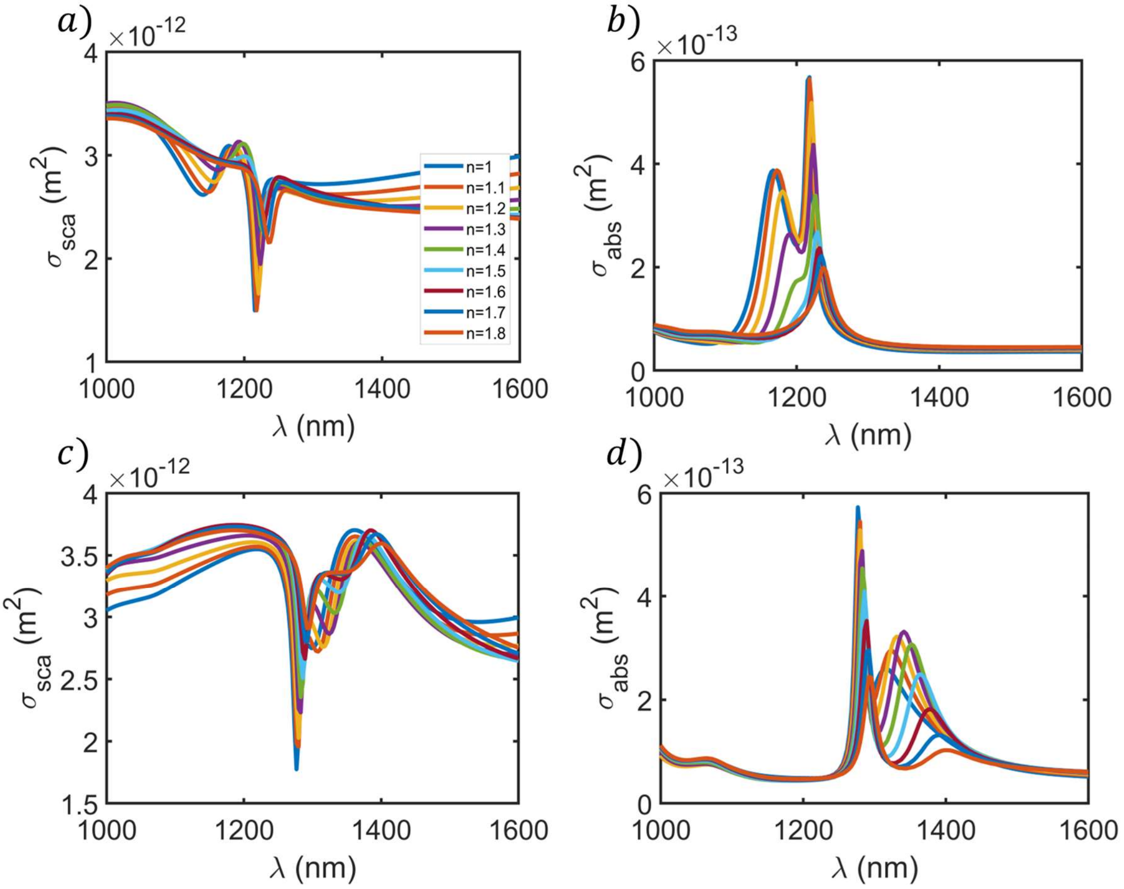

Figure S12. Scattering and absorption cross sections for a gold/silicon hybrid structure with $d = 0$ nm, $l = 290$ nm for various substrate refractive index values. a), b) cross sections for $h = 155$ nm and c), d) for $h = 200$ nm.

Figure S12 shows the scattering and absorption cross sections for a set of values of the substrate refractive index, from $n = 1$ (isolated structure) to $n = 1.8$ (near to the alumina substrate) in 0.1 steps. All the representations show a similar trend, with the generated modes redshifted, as expected. This redshift can be explained in terms of the well-known image charge problem.

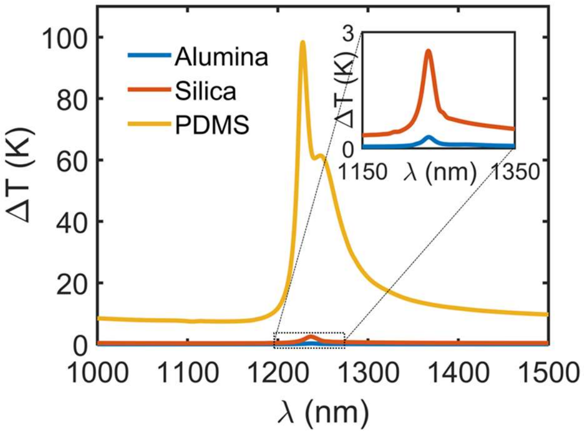

Figure S13. Temperature increment spectra for the hybrid GaAs-Gold disk/ring on the three different substrates: alumina (blue), silica (red) and PDMS (yellow)

In Figure S13, the spectral thermal response of the three substrates is obtained. All curves show the spectral resonance at similar wavelengths as the considered materials have similar optical properties (the influence of the substrate refractive index on the architecture cross sections is shown in Figure S12). However, they present radically opposed thermal responses as a consequence of their thermal conductivity contrast. Thus, a well-defined trend can be extracted from the maximum temperature increment. As the thermal conductivity increases the maximum temperature reduces.
